# Mentorship, meritocracy, and scientific journal reviewing

**DOI:** 10.1016/j.jdin.2021.04.003

**Published:** 2021-05-04

**Authors:** Jonathan Kantor

**Affiliations:** Department of Dermatology, Center for Global Health, and Center for Clinical Epidemiology and Biostatistics, University of Pennsylvania Perelman School of Medicine, Philadelphia, Pennsylvania; Florida Center for Dermatology, PA, St Augustine, Florida

As the Journal approaches the 1-year anniversary of its first published issue, I would like to thank the many editorial board members, contributors, and reviewers who have helped make *JAAD International* not only a reality but a success. I would also like to acknowledge the energetic and tireless support of the team at *JAAD* (led by Dirk Elston and Jane Grant-Kels), the Academy, and Elsevier. Indeed, no one could have foretold that such a dramatic success and such a meteoric rise would have been possible, and certainly, to have experienced such growth and support in the context of a pandemic is all the more remarkable.

Although no journal can exist without submissions, no high-quality journal can exist without excellent reviewers. As such, it is worth highlighting the critical role played by reviewers in the success of not just *JAAD International* but every other scientific journal as well. Reviewers work anonymously and tirelessly, and they take time from their own busy careers to vet the work of others and provide words of support, advice, and criticism. Our reviewers agree to donate their own valuable time and energy to help the scientific enterprise, and some of our most prolific reviewers accept requests to review on a shockingly frequent basis.

What makes a great reviewer? Subject matter expertise can be critical; however, particularly with the rise of indexing systems, many outstanding reviewers are also generalists, with a wide breadth of knowledge and a keen eye for detail. Given the clinical focus of *JAAD International*, reviewers with experience in epidemiology, study design, and biostatistics are often called on to make important contributions. The most important qualification, however, is a willingness—and even an eagerness—to engage with the scientific community, read closely, and provide honest input.

As the demands on our time only continue to increase, it can be difficult to prioritize reviewing—an unpaid, thankless, and time-consuming endeavor. Yet reviewing is also in some ways the most prosocial act that we can perform as physicians and scientists, and in its anonymity, there is also the opportunity to do the right thing to advance our field forward.

Reviewing also provides an opportunity for mentorship, both formal and informal. In addition to the existing mentorship programs available through the *JAAD* journals, I am always happy to provide feedback to reviewers on request; just as reviewing is a prosocial act, so is the mentorship that can sometimes be helpful in taking a reviewer from good to great—or from great to world-class.

Reviewing articles—and particularly reviewing articles well—forces the reviewer to read closely, pause, and consider the larger questions at hand. As such, this yields reviewers who are better readers and writers of the scientific literature.

I encourage all of our readers to consider joining our outstanding team of reviewers. There can be no peer review without peers who review. Thank you again!

## Conflicts of interest

None disclosed.

